# Palestinian Muslim College Students’ Attitudes to Mental Health Treatment: A Comparative Study

**DOI:** 10.3390/ijerph192316005

**Published:** 2022-11-30

**Authors:** Wahiba Abu-Ras, Amir Birani, Zulema E. Suarez, Cynthia L. Arfken

**Affiliations:** 1 School of Social Work, Adelphi University, Garden City, NY 11530, USA; 2 Clinical Social Work, Therapist Daliyat AL-Karmel, Daliyat Al-Karmel 3005600, Israel; 3 School of Social Work, Loyola University, Chicago, IL 60660, USA; 4 Department of Psychiatry and Behavioral Neurosciences, Wayne State University, Detroit, MI 48201, USA

**Keywords:** culture, religiosity, mental health, Muslim college students, occupied Palestinian territory, Israel

## Abstract

This study examined the association between the degree of religiosity, combined with cultural beliefs, social stigmas, and attitudes towards mental-health treatment in two groups, who, despite having similar cultural and religious affiliation, have experienced different socio-political contexts: Palestinian Muslim college students living in the Occupied Palestinian Territory (OPT) and Israel. The study was guided by Tanhan and Young’s (2021) conceptual framework. Methods: A snowball recruitment strategy was applied, using a cross-sectional survey. A total sample size was 214 students, 105 from the OPT and 109 from Israel. Results indicate that students from the OPT (*n* = 105) did not differ from those living in Israel (*n* = 109) on religiosity using the Islamic Belief scale, or Attitudes Towards Mental Health treatment (*F*(1, 189) = 1.07, *p* = 0.30). However, students from the OPT had higher confidence in mental-health professionals (*M* = 15.33) than their counterparts (*M* = 14.59), and women had higher confidence (*M* = 16.03) than men (*M* = 13.90). The reliance on traditions for Muslim students over Western mental-health approaches is a critical factor in predicting the attitudes towards students’ mental problems and their chosen treatment. Sociopolitical context played a significant role in shaping attitudes toward mental-health providers.

## 1. Introduction

There is a growing concern about mental health on college campuses. Although college is a time of rapid intellectual growth and social development, it also involves changes in many areas, such as personal, social, and institutional environments [1]. Many mental health issues emerge during late adolescence and early emerging adulthood, including anxiety, insomnia, and depression [2,3]. Despite the added stressors and transitions during college, students often avoid seeking professional help [4].

Mental health issues are particularly prevalent among students of diverse racial/ethnic and religious backgrounds who encounter stressors when moving to a secular world different from their home [5,6]. However, for students studying and living in politically unstable environments, suppressing their mental-health needs, quality of life, and well-being is a form of coping [7,8,9,10]. This is especially true for Muslim students who adhere strongly to their religious practices and beliefs and use religion to cope with adversity and stress [11]. Thabet et al. [12] found that Palestinian college students had high levels of death anxiety. The authors attributed these results to recurrent exposure to war trauma and the ongoing political conflict in the area. Beyond discrimination, Palestinians studying in Israeli universities also face acculturation stresses and the inherent conflict of preserving Arab aspects of their cultures while including Israeli elements [13]. These added stressors may impact their mental health and attitudes toward professional counseling services. Giacamen et al. [10] report that Palestinian Muslim students in the OPT are likely to be more stressed than their Israeli counterparts because of higher political conflict and instability.

This study is the first attempt to compare attitudes towards mental-health providers and service use between two samples of Muslim Palestinian college students in the OPT and Israel.

### 1.1. Attitudes toward Mental Health Treatment

Palestinians’ perceptions and attitudes toward mental health treatment are associated with help-seeking behaviors [14]. Stigma, cultural values, religious beliefs, religiosity, and lack of resources pose significant challenges to seeking help from a mental-health professional [15,16,17,18,19].

### 1.2. Stigma

Stigma about mental-health issues is universal. However, how stigma is framed, practiced, and perceived may differ across different cultures [15]. Race/ethnicity, religion, gender, and political climate are important factors that predict help-seeking attitudes among college students. For example, Asian Americans hold negative attitudes toward mental-health services and are less likely to seek psychological counseling due to perceived self-stigma than Caucasians [20].

Stigma among Arab and Palestinians is firmly attached to mental illness, preventing many people from receiving mental-health treatment [14,21,22]. A systematic review of 34 studies conducted in 16 Arab countries revealed stigma related to mental-illness treatments and professionals [23]. Another qualitative review of mental illness in the Arab world shows that stigma toward mental illness persists and is beyond socio-cultural barriers [24,25]. Another study suggests religious Arab Muslims may hold more stigma and negative attitudes toward mental illness than secular groups [26].

Stigma is a crucial determinant of students’ help-seeking behaviors and attitudes toward mental-health treatment [27,28], along with gender [29] and cultural and religious beliefs [22,30,31]. Soheilian and Inman [32] found a strong relationship between self-stigma and attitudes toward counseling among Middle-Eastern Americans. The latter tend to internalize the prejudices they experience in their cultural and familial environments.

### 1.3. Religious Values

In addition to stigma, cross-cultural studies suggest that religious values are the most common factor affecting Muslim attitudes toward mental-health issues [14]. Stigma, religiosity, and attitudes toward treatment deter Arab Americans [33] and Middle East-based Muslim college students from mental-health-care use [34,35,36,37].

Herzig et al. [38] examined the relationship between stigma tolerance and religiosity in active coping among Muslim-American college students. The findings suggest that religiosity positively correlates with active coping in this group, fully mediated by using religious-coping strategies [39]. Studies consistently identify religiosity, both in beliefs and practice, as a protective factor [40,41]. Hence, prayer and religiosity become essential for coping with mental distress [42]. However, religiosity may be a barrier to seeking professional mental help; more religious clients expressed greater distrust towards healthcare systems and were more concerned about the social stigma associated with seeking treatment [43,44].

Several studies in Western countries have identified discrimination patterns based on ethnicity and religion regarding mental-health issues among Arabs living in the U.K. [27] and the USA [42,43,44]. These studies also revealed that Arabs prefer seeking faith healers or turning to God as the first approach to treating mental illness. Similar findings were consistent among Arabs and Muslims living in Western societies, who often turn to their religious leaders for psychological help and avoid seeking formal mental-health services [14,39,43,44].

### 1.4. Discrimination and Lack of Resources

Research also shows a strong relationship between discrimination and poor mental and physical health among minority Arabs in Israel [40]. In addition to anticipated stigma, individuals with mental illness often experience feelings of rejection and discrimination by society, which contributes to self-esteem loss, social withdrawal, depression, a decrease in asking for help, and a lower quality of life [41]. Lipson et al. [5] reported that 46% of Arab/Arab American students and 57% of multiracial students did not seek help because “providers are not sensitive enough to cultural issues” [5] (p. 351).

For example, Palestinians experience discrimination through Israeli government policies and face exclusion, challenges of integration, and inequality [42]. Like other minorities, Muslim Palestinians in Israel tend to utilize mental-health services much less than those from dominant cultural groups [34]. Although Palestinians in Israel are supposed to be served by the same Israeli healthcare system, they still face interpersonal and institutional discrimination [43]. In addition, inadequate mental-health care may lead to delayed help-seeking behavior and reluctance to seek mental-health treatment [44].

Palestinian students at Israeli universities experience stress and hardship due to a lack of cultural and religious sensitivity on campus [43,45]. Therefore many of the Palestinian population in the OPT need mental-health interventions.

Nevertheless, mental-health services provided by the Palestinian Ministry of Health are under-resourced, ineffective, inefficient, and unavailable [46,47,48,49,50]. Similarly, ethnic inequality in healthcare and marginalization heavily influences the mental health of Palestinians in the OPT [50,51]. Finally, it may be necessary to investigate the relationship between demographic variables and stigma. The influence of stigma and negative attitudes toward mental-health issues [52,53] is more substantial among male than female students [54]. Several studies have found that women are more likely to have a positive attitude toward mental health help than men [19]. Positive attitudes are also associated with gender and age. In their study, Mackenzie et al. [55] found that older adults are more likely to seek help than younger adults. These findings suggest that a lack of psychological openness is associated with negative attitudes in men, which may explain the underutilization of mental-health services. In addition, Amin et al. [56] argue that socialization and masculinity norms endorsed at an early age may also shape male and female attitudes toward mental-health help-seeking behaviors.

### 1.5. Social and Family Support

Social and family support is a psychosocial resource that can reduce the negative effects of stressful life experiences on health [57]. Within collectivist or group-oriented societies, the family provides the primary support and nurturance of the individual. Community and familial resources belong to group members for familial or communal benefit. According to Arnault [58], health is not an individual asset but an outcome and resource of the well-functioning group under the group-oriented cultural model. Conversely, an illness reflects poor functioning that hurts the group. According to a cross-national comparison of Middle-Eastern University Students, ethnic populations seek help from non-medical providers, such as family, friends, and religious institutes [14]. Arab college students seldom take advantage of available mental-health services due to alternatively relying on social and family support systems [14,59,60].

Similarly, Jafari [61] and Pines [62] found that Palestinian-Muslim youth and adults in Israel prefer to seek help from informal and kin networks, such as parents and friends, before turning to healthcare professionals, such as doctors, educational counselors, or psychotherapists. Turning to the informal support system is usually perceived as normative, available, and more understanding of their needs, while seeking professional help is more stigmatizing [42,63]. These cultural practices can deter mental-health services use among Arab college students during stressful times.

### 1.6. Palestinian Muslim College Students in a Socio-Political Context

Enrollment rates in higher education in the Middle East and North Africa are slightly higher than average, with current rates at 31%, compared to the worldwide rate of 30% [64]. Middle-Eastern college students are likely to engage in politics, which they combine with a sense of nationalism [65,66]. Palestinian college students expect to actively build their country politically through their future professional work [17,18]. For many Palestinian students, attending higher education has a political sentiment and value. The cultural imperative in Palestinian society is “not just to become educated but also to use that education for the benefit of Palestinian liberation” [67] (p. 64). In addition to their stressors as students, they perceive a political responsibility to resist and fight oppression and occupation. The exposure to prolonged political conflict between Israel and Palestine has a lasting impact on the Palestinian people’s well-being and other aspects of their lives. Due to the protracted toxic socio-political context in the region, Palestinian people have the highest burden of mental disorders in the Eastern Mediterranean Region and globally [50]. A growing body of evidence suggests an inter-relationship between political violence, humiliation, and adverse mental health outcomes [10,50], such as acute and complex PTSD, emotional and behavioral problems, and depression symptoms among children and adolescents [68].

Despite the OPT’s ongoing political unrest and economic hardships, Palestinians have a 96% literacy rate. About 53 accredited post-secondary education institutions in the OPT offer degrees in over 300 fields to 44,446 graduate students. Most students are female: 27,035 females vs. 17,411 males [69].

Palestinian citizens in Israel constitute 21% (2 million) of Israel’s total population. The vast majority (83%) are Muslims, 9% are Druz, and 8% are Christians [70]. Over 300,000 Palestinian Arab students are currently pursuing academic degrees in Israeli universities. About 16.1%, 13%, and 6.3% are in bachelor’s, master’s, and doctoral degrees, respectively, making 78.5% in all forms of higher education [71]. Although Palestinian Arabs in Israel share the same religion and culture as those in the OPT, they are considered a cultural and religious minority.

Considering the unique political climate and the increased risk of mental-health concerns among Palestinian-Muslim college students in both parts of the region, identifying the factors that explain their attitudes toward mental-health services is a pressing issue. Therefore, examining the relationship between variables, such as religiosity, cultural values, and Palestinian-Muslim students’ attitudes toward mental-health professionals and treatment is essential.

## 2. Theoretical Framework

**Figure 1:**
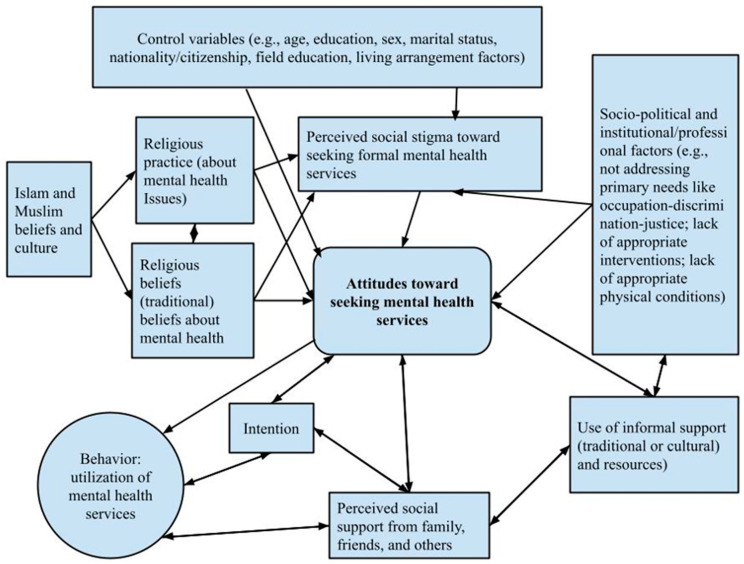
The Concept Map. Adapted from Tanhan and Young [72] with permission.

This study is guided by Tanhan and Young’s Contextual Theoretical (Conceptual) Framework [72]. In this model, individual and contextual factors are predictors of human behavior and, in this case, the use of mental-health services. Accordingly, Tanhan and Young drew on the theories of Planned Behavior and Reasoned Action (see conceptual map, Figure 1). In response to other researchers’ calls to examine Muslim individuals’ proclivity to use mental-health services, they developed and empirically validated this framework.

Individual variables include cultural beliefs about mental-health problems and their causes. Other components in the framework include background variables (education, gender, race/ethnicity, and economic factors), knowledge about formal mental-health services, attitudes towards seeking formal mental-health services, perceived social stigma toward seeking mental-health services, perceived behavioral control toward seeking mental-health services, and intention (readiness or openness toward seeking mental-health services as predictors of use of mental-health services. Our study included all the above factors except “perceived behavioral control toward seeking mental-health,” which was replaced with “perceived social support” as a possible cultural and contextual factor. Finally, we collected data in two different geographic and political locations to control for mezzo and macro-environmental factors on the need for and the use of mental-health services.

### 2.1. Objectives of the Study

The study has three major primary objectives: (a) To examine the attitudes toward mental health and mental-health services among Palestinian-Muslim students in the OPT and Israel; (b) to explore the differences between the two groups in levels of religiosity (practice and beliefs) and attitudes toward mental health and mental-health services, and (c) to assess the relationship between religiosity and attitudes toward mental illness and treatment-seeking of Muslim students in both in both groups.

### 2.2. Hypotheses

The research has two primary hypotheses: (a) There is a significant difference between attitudes towards mental-health concerns and treatment between groups after controlling for religiosity, and (b) there is a significant difference in attitudes toward mental-health concerns and treatment between groups when controlling for age, sex, marital status, living arrangement, the field of study and self-rated health.

## 3. Materials and Methods

### 3.1. Participants and Recruitment

Muslim students from Israel and the OPT currently enrolled in university studies were eligible for the survey, regardless of sex or age. A snowball recruitment strategy was used, with the survey initially distributed to potential Muslim students in both areas by local recruiters known to the second author. The second author then completed a follow-up telephone interview with 30 students from the OPT and 40 from Israel for quality-assurance purposes. All recruiters attended training on appropriate and ethical recruitment methods and techniques, the study objectives, participant rights, potential risks, and confidentiality issues. The local recruiters distributed a print copy of the survey. The survey was anonymous, and no identified personal information was collected from either group. The research assistant completed the questionnaire on the recruitment sites (OPT or Israel). This study was approved by the Institute of Research Board at the Ben-Gurion University of the Negev.

### 3.2. Measures

The survey included demographic questions and previously validated scales. The demographic information comprised age, gender, marital status, living arrangement, field of study, self-rated health, and socioeconomic status (SES). Self-rated health was measured on a scale from 1 = Very Good to 4 = Poor. SES was also self-rated on a scale from 1 = Very Good to 5 = Poor.

The Attitudes toward Seeking Professional Psychological Help Scale [73] consists of 29 items answered on a 4-Likert scale (0 = Disagree, 3 = Agree) with four subscales: recognition of need for psychotherapeutic help, stigma tolerance, interpersonal openness, and confidence in the mental health practitioner. Scores range from 0 to 87, with higher scores indicating more positive attitudes toward seeking professional psychological help. For stigma tolerance, a higher score meant more tolerance, consistent with a more positive attitude. Internal consistency of the scale has ranged from 0.79 to 0.82 [74]. There is also evidence from diverse populations on concurrent validity and internal consistency (e.g., Asian Americans [75]; Muslim adults in Toledo, Ohio [76]; and African, Asian, and Latin-American international students [77]).

The Multidimensional Scale of Perceived Social Support (MSPSS) was developed by Zimet, [78]. This scale was used to assess the students’ perception of social-support levels from families, friends, and special friends. It contains 12 questions, each with a 7-point response option (from 1 = “Very Strongly Disagree” to 7 = “Very Strongly Agree”). The total score ranges from 12 to 84, with higher scores reflective of greater perceived social support. Several studies have demonstrated good reliability, with internal consistency ranging from 0.84 to 0.93 [21,22,79].

The Religiosity of Islamic Scale (RoIS) developed by Jana-Masri and Priester [80] is an 18-item tool used to assess two subscales: Islamic Beliefs (IB with 8 items) and Islamic Behavioral Practices (IBP with 10 items). The developers reported good internal consistency of α = 0.66 for the IB subscale and α = 0 0.66 for the IBP subscale. The IB was answered using a 5-Likert scale, ranging from 1 = “Always” to 5 = “Never”, and the IBP was answered using a 7-Likert scale ranging from 1 = “Strongly agree” and 7 = “Strongly disagree.” Five items on the original scale were reverse-scored. Lower scores indicate greater religious practice and beliefs. One item in the IB subscale was modified for this study to read: “I believe that a man can marry more than four women” (reverse-coded). Examples of the IB items included: “I believe that the Qur’an is the final word of Allah,” and the IBP items included “I pray five times a day”.

### 3.3. Analysis

Before analysis, responses to the survey were examined for missing data and outliers. Scales were constructed with particular attention to internal consistency for the total sample and each recruitment site. Internal consistency for ATSPPHS was α = 0.76 for the total. However, the subscales had internal consistencies that were less than adequate (range of α = 0.44 to 0.57). MSPSS and the subscales had internal consistencies greater than α = 0.80. For IB, the internal consistency was adequate for the total sample (α = 0.60) but higher for the Israeli sample (α = 0.73) than for the OPT sample (α = 0.42). In contrast, the IBP had less than adequate internal consistency (α = 0.51). Individual items were examined for the IBP but deleting individual items did not improve the internal consistency. Deleting individual items for the IB led to higher internal consistency for the Israeli sample but not for the OPT sample.

Bivariate analysis was then conducted examining the association of recruitment site with demographic, health, and social-support variables using Χ^2^ for categorical variables, *t*-tests for continuous variables, and Mann-Whitney tests for the ordinal variables of SES and self-rated health. When the assumption of equal variances was not met, *t*-tests with an unequal variance approach were used. Associations between the continuous measures of age, attitudes, social support, and religiosity were examined using Pearson correlation.

Finally, multivariate analysis of variance models was used to test the a priori hypotheses that recruitment location would remain significant in predicting attitude independent of demographic or religiosity. Estimated means were calculated. No interactions were included in the model.

## 4. Results

### 4.1. Sample Description

The final sample comprised 105 Muslim students from Israel and 109 from the OPT. Most of them were females (62.2%, *n* = 139), single (85%, *n* = 182) with a mean age of 24.41 (SD = 4.03) but ranging from 20 to 48 years old. When asked to report their socioeconomic status (SES), 38% of the students (*n* = 81) identified as middle SES and 35.2% (*n* = 75) as good. Most participants (72.9%, *n* = 156) described their health as “very good.”

The mean score on the ATSPPHS was 44.81 (SD =9.29), reflecting that, on average, the respondents were evenly divided between agreeing and disagreeing towards questions on seeking professional psychological health support. For the subscales, stigma tolerance had the least support (*M* = 5.19 or *M* = 1.04 on each item), followed by confidence (*M* = 15.32 or *M* = 1.70 on each item), interpersonal openness (*M* = 12.48 or *M* = 1.78 on each item), and recognition of need (*M* = 15.50 or *M* = 1.94 on each item).

The MSPSS had a mean of 61.37 (SD = 15.21), a score reflecting on average that the respondents mildly to strongly agreed with each social-support question. In ranked order, the scores on social support from a special friend (*M* = 21.46) were highest, followed by social support from family (*M* = 20.88) and then social support from significant others (*M* = 18.99), although they were all high.

For the Islamic Belief subscale, the mean of the scale was 20.07 (SD = 7.54), a score that indicated that, on average, the respondents agreed to somewhat agreed with each Islamic belief question. For the Islamic Belief Practice subscale, the mean was 24.71 (SD = 5.14), indicating that, on average, the respondents sometimes agreed with each practice. The item with the lowest agreement was “I give charity money”.

### 4.2. Bivariate Analysis

For the entire sample, the Islamic Belief and Belief Practice subscales were correlated (r = 0.61, *p* < 0.001), suggesting that, although related, they measure different aspects of the students. The subscales of social support also showed significant correlations (r < 0.40), such that people who reported strong social support from one source were more likely to report strong social support from the other two sources. However, the subscales of attitude either were not correlated or weakly correlated (r < 0.40). Most of the correlations between domains were very small (r < 0.15).

In other bivariate analyses, living with parents was associated with lower scores on the Islamic Beliefs scale than living in dormitories or rented apartments (*M* = 20.54 versus *M* = 17.19, *t*(212) = 2.28, *p* = 0.024). Women were also significantly more likely than men to live with parents (89.9% versus 77.5%, Χ^2^ (1) = 5.96, *p* = 0.015).

Other comparisons by gender revealed that women had higher ATSPPHS scores (*M* = 64.71 versus *M* = 59.61, *t*(206) = 3.40, *p* < 0.001), confidence scores (*M* = 16.17 versus *M* = 13.75, *t*(206) = 3.38, *p* < 0.001), more stigma tolerance (*M* = 5.80 versus *M* = 4.80, *t*(206) = 2.53, *p* = 0.012), and more support from a special friend (*M* = 22.40 versus *M* = 19.55, *t*(208) = 3.05, *p* < 0.001) than men. Gender was also associated (X^2^ (3) = 24.65, *p* < 0.001) with the field of study, with over twice as many men studying physical or biological sciences as women (39.4% versus 11.6%) and almost three times as many women studying Humanities (20.3%) as men (7.0%).

### 4.3. Differences between Muslim Palestinian Students in the Occupied Palestinian Territory and Israel

Examining our main hypotheses, several differences appeared between the samples of OPT and Israeli students. Table 1 shows that the OPT sample was older, more likely female, and more likely to live with their parents than the Israeli sample. They also differed in what they were studying at the university.

Table 2 shows that the ATSPPHS did not differ significantly between OPT and Israeli students, being low for both groups of students. The effect size for the observed nonsignificant difference in ATSPPHS was 0.14, conventionally considered as less than a small effect size. For the subscales, Confidence was significantly different by recruitment site, with higher mean scores in the OPT students than in the Israeli students. The effect size was 0.39, a small to medium effect.

The two groups of students also differed in their reports of total social support, especially social support from families, with students in Israel reporting both greater total social support and social support from families than students from the OPT.

### 4.4. Multivariate Analysis

A multivariate analysis confirmed that the ATTSPPHS Total score did not differ based on recruitment site, even when sex, the independent and dependent variables, were included in the model.

Multivariate analysis with Confidence as the outcome variable was conducted in exploratory analysis with backward selection using gender, age, a field of study, living in a village, and living arrangement. After elimination of clearly non-significant factors (*p* > 0.40), recruitment site was still not significant (*F*(1,189) = 1.07, *p* = 0.30) but gender (*F*(1,189) = 8.13, *p* = 0.005) and field of study (*F*(3,189) = 2.75, *p* = 0.044) were significant in the final model (*F*(6,189) = 4.49, *p* = < 0.001). The estimated means showed OPT students had higher confidence (*M* = 15.33) than Israel students (*M* = 14.59), women had higher confidence (*M* = 16.03) than men (M = 13.90), and Social Science students had the highest confidence (*M* = 16.05), followed by other studies (*M* = 15.71), then Humanities (*M* = 14.26), and the lowest among Physical and Biological Science students (*M* = 13.83).

The total score on social support was also modeled in the exploratory analysis. For the model with total social support as the dependent variable, recruitment site (*F*(1,207) = 8.16, *p* = 0.005) and sex (*F*(1,207) = 5.01, *p* = 0.026) were significant, clearly indicating the observed difference in social support was not due to different gender distribution by recruitment site. The overall model was significant (*F*(2,107) = 5.75, *p* = 0.004). The estimated means showed that Palestinian students in Israel reported higher support (*M* = 63.61) than OPT students (*M* = 57.65), and women reported higher support (*M* = 63.10) than men (*M* = 58.16).

## 5. Discussion

Using an adaptation of Tanhan and Young’s Contextual Theoretical (Conceptual) Framework [72], this study examined differences in attitudes toward professional mental-health care in two groups of Palestinian-Muslim college students recruited from different socio-political contexts: OPT and Israel. The results showed no difference in attitudes between the two groups based on sites. The attitude score showed that the sample had a positive view of seeking and receiving professional psychological treatment for mental-health problems. On religiosity, they mostly agreed with the statements on the scale, and most of them lived with their parents, suggesting that the sample is not rejecting their culture. They are also generally healthy and from the middle class. Compared to their Israeli counterparts, OPT respondents expressed a higher level of confidence in mental-health professionals, which corresponded with a previous study [14] indicating that Palestinian participants were higher in “confidence in mental health,” followed by Israeli Arab, Egyptian and Kuwaiti subjects consecutively. According to our adapted conceptual framework, this may be due to alienation from the Israeli mental-health system and the importance of context when analyzing mental-health care use. These findings correspond to previous studies indicating that cultural sensitivity is significant in the therapeutic environment both institutionally and in therapeutic relationships [81,82,83], particularly concerning exposure of personal feelings toward a stranger [62,63,64]. Since most therapists serving in the OPT are Palestinians, students may feel more comfortable consulting co-ethnic professionals who share their culture, religion, language, and sociopolitical context.

Mental-health interventions in Palestine are also influenced by contextual political conflict and occupation, and the primary approach used is based on community provision care [51]. Therefore, the primary goal of counseling services in Palestine is to promote social empowerment through guidance on social issues and human rights in a broader social context rather than on the individual level [84]. Accordingly, Palestinian college students from the OPT may not hesitate to seek help. They may consider counseling and support services to strengthen their personal qualities and empower themselves and their communities. This view is consistent with a cultural model proposed by Arnault [58,85] that “provides specific and consensual guidelines about ideals, values, motivations, goals, social roles and preferred social behaviors’’ [85] (p. 3). It helps people, such as Palestinians in the OPT, to direct their help-seeking behavior toward maintaining wellness and easing distress.

### 5.1. Confidence in Mental Health Providers

As noted above, Palestinian female college students in Israel may have less confidence in mental-health professionals due to the structure and culture of the service-delivery context and the political environment within which these institutions operate. Previous and current studies [86,87,88] show that the Palestinian-Israeli conflict negatively impacts the therapeutic relationship between Israeli providers and Arab Palestinian patients. A second interpretation of the lower confidence in professional mental-health services among Palestinian students in Israel is that students may also face cultural and structural barriers to accessing counseling and psychotherapy [40,42].

Furthermore, another reason is that most providers of mental-health services are Israelis or use the Eurocentric approach. Because Israeli universities, campuses, and culture adhere to Western democratic values, mental-health services are Eurocentric. Israeli-Jewish providers and supervisors encounter cultural differences with Arab supervisees and patients, negatively impacting confidence building in the working relationships [89]. Therefore, Palestinian Muslim students studying at Israeli universities may perceive and distrust Israeli professional services as part of an oppressive colonial system with limited cultural sensitivity towards them [40,42].

These factors and the Palestinian cultural predisposition to seek help from family and their informal network may foment internal conflict and ambivalence about formal Israeli mental-health services [62,63,64,65,66,90]. Friends and significant others are also seen as more available and understanding of their needs [42]. Given these findings, cultural context is particularly substantial when dealing with mental-health problems.

### 5.2. Religious Values

The present study showed that the two groups did not differ in religiosity and did not find an association between religiosity and attitudes toward mental-health treatment. This unexpected finding may be due to the low internal reliability of the measures, especially in the OPT sample.

However, both groups had significant differences regarding their confidence in professional help. Palestinian students from the OPT majoring in social sciences expressed more positive attitudes towards and confidence in mental-health professionals than their Palestinian counterparts [91,92]. They found that Social Science students were more socially sensitive than students majoring in STEM areas. Also, students who focused on Social Studies and Humanities expressed more willingness to seek psychological help than other students. Another interpretation of these results is that most OPT mental-health services consist of the community provision of care [49], which is usually less stigmatized than psychiatric hospitals and clinics. Also, mental-health professionals are part of the Palestinian population and share the same unstable political and cultural environment that is hardly conducive to developing a sustainable mental-health system [10].

Palestinian-Muslim students from Israel majoring in Social Sciences and STEM areas are more likely to be influenced by their Jewish student counterparts’ negative attitudes, the political climate, and other national events in or outside Israel. For example, Al-Krenawi et al. [14] found that Palestinian students from Israel reject the Israeli-Jewish majority society, lacking confidence in professional help, as they feel discriminated against by the services offered. Therefore, they express less confidence in mental-health professionals than their Palestinian counterparts in the OPT. Besides, Vogel et al. [93] found that when the public holds negative attitudes and treats professional psychotherapists as unacceptable, it directly affects the individual’s attitudes and creates a negative stigma towards the proposed service.

In both groups, gender was a predictor of differences in attitudes toward mental health and seeking professional treatment, with women having more positive attitudes than men. According to other studies [55,94,95], Arab women report more positive attitudes toward seeking help than men. In contrast, Leshem et al.’s [96] study of Palestinian adolescents found that girls were less likely to seek mental health care than boys. This hypothesis relates to the change in women’s attitudes in both groups. Furthermore, women’s positive attitudes are likely influenced by the reported lack of family support they receive compared to male participants, the increased responsibility they may have toward their families, and their adherence to cultural values [97]. In addition, the expectation that men are strong and independent may prevent them from seeking counseling, regardless of self-disclosure benefits [43]. Help-seeking behaviors and attitudes are deemed incongruent with socialized masculine norms. These findings contradict other research claiming that Palestinian women, including Palestinian-Muslim female students from Israel, cope with changes in the modernization process while wavering between conformity and traditional values [98]. Despite the relative improvement in Palestinian women’s status in Israel compared to Palestinian women from the OPT and the increase in college attendance, there is still a traditional divide within the family that maintains power balances, dominant patriarchy, and male supremacy [99,100]. Most Palestinians in the OPT and Israel do not recognize women’s efforts in the labor force, nor do they count as part of the household income. Educated and career women are still regarded in Arab cultures as caregivers who are expected to adhere to patriarchal values and comply with their demanding professions [101].

### 5.3. Limitations

This study has several limitations. The data are not generalizable, as they consist of a non-random convenience small sample from a limited number of universities, accessible and known to recruiters on both sides of the country. Most students are also healthy and come from the middle class. Respondent bias is a possibility, especially since this study was conducted during a time of intense political conflict in the region, which could alter some results. Despite these limitations, findings indicate significant differences in attitudes towards mental-health professionals based on their level of confidence and stigma tolerance between the two groups.

## 6. Conclusions

The results indicate no difference in both groups’ perceptions of receiving help from mental-health professionals. However, the level of confidence toward mental-health professionals, stigma tolerance, and perceived support from friends did differ due to differences in gender distribution and fields of studies. The findings show that culture, religion, tradition, and context are consistent determinants of attitudes toward mental health-seeking behavior. Although both Israeli and OPT students had positive attitudes towards seeking professional mental health, women’s confidence in mental services differed according to geographic location, with the former being more hesitant to seek help. Therefore, relying on tradition for Muslim students over Western mental-health approaches is critical in predicting attitudes toward students’ professional concerns and their treatments. The failure to identify, acknowledge, or treat mental illnesses within the cultural context is likely to prolong mental health afflictions in these young people [96].

Consistent with previous research, gender is an important background variable in understanding mental-health services-seeking behavior. Compared to men, women are more positive about treatment and confident about discussing their mental-health issues with professionals, especially when family support is lacking. This may also be due to their expression of greater need. Having greater needs may motivate them to seek help.

Therapeutic relationships in conflict regions worldwide are complicated by political conflict [97]. For Muslim Palestinians living in Israel and the OPT, a lack of confidence in mental-health providers could be due partly to the political conflict or inefficient monitoring mechanisms within the healthcare setting. Thus, it is important to address the issue of political sensitivity among mental-health providers by understanding their client’s needs and cultures and using cultural humility to promote strong relationships with them. There has been some progress in integrating mental-health services into primary health-care in the Arab society in Israel. Still, more qualified Arab professionals are needed to reduce stigma and build a more trusted relationship between providers and patients. This study suggests that more cultural humility training programs are needed, especially inside Israel and the OPT, where Israeli and Palestinian providers work together. In these training programs, providers, patients, and students learn to identify, recognize, accept, and respect each other’s emotions about their conflicting political views and stands.

### Future Direction for Research

The study results suggest important directions for future research, which can inform the development of culturally responsive mental-health intervention and prevention programs that meet the needs of this specific population. Although we did not directly empirically validate Tanhan and Young’s Contextual Theoretical Framework [72], our findings support the importance of contextual and cultural variables, such as geographic location, political environment, and adherence to cultural values. In the case of college students, large-scale research and in-depth exploration of the intersectionality of student identities, religiosities, and mental well-being during political conflicts is needed. As the geopolitical conflict between Israelis and Palestinians affects mental-health treatment among Palestinian Muslim College students, it may explain the lack of mental-health services used in Israel and the OPT.

Furthermore, Israeli society adheres to Western codes and is not as culturally responsive to Palestinians’ mental-health needs. Muslim students in the OPT are more likely to accept receiving psychotherapy than their counterparts. However, it is still less valuable for cultural reasons, as help-seeking from friends, family, and significant others is valued and encouraged more than professional mental-health services. Thus, social workers and mental-health providers should know the interrelationship between religious beliefs and traditions when helping Muslim students and attempt to bridge the gap between formal and informal help-seeking behaviors. To that end, service providers should engage students, imams, and other supportive community leaders when designing culturally syntonic mental-health services based on cultural conceptions of mental-health issues. This participatory process would foment trust, agency, and empowerment among a historically oppressed population. Culturally insensitive service provision is a serious concern for both groups. It is vital for Muslim students in Israel and the OPT, where most mental-health services and approaches are Western-based. Help and change should be titrated to each area’s underlying needs. These recommendations can also benefit the Muslim community by reducing stigmas, improving awareness, and increasing access to mental-health services.

Research should examine what programs and mental-health resources colleges use to improve access for Palestinian-Muslim college students. Furthermore, a thorough assessment is needed regarding how these existing resources correlate with increased access to and use of mental-health services and how culturally sensitive these resources are.

## Figures and Tables

**Table 1:** Demographic, social, and educational Characteristics by recruitment site.

Characteristics	OPT		Israel		t(df) or Χ^2^ (df)	*p* Value
	(*n* = 109)		(*n* = 105)			
	M	SD	M	SD		
Age	25.29	4.83	23.48	2.71	3.30 (161.55)	<0.001
Socioeconomic status	2.39	0.91	2.55	0.94	1.21 (211)	0.23
	*n*	%	*n*	%		
Gender					4.79 (1)	0.029
Males	28	26.7	43	41		
Females	77	73.3	65	59		
Marital status					2.01 (1)	0.16
Single	89	81.7	93	88.6		
Married	20	18.3	12	11.4		
Residence of birth					3.59 (1)	0.06
City	34	32.1	47	44.8		
Village	72	67.9	58	55.2		
Living arrangement					10.64 (1)	<0.001
With parents	102	93.6	82	78.1		
Dorms/rent apartment	7	6.4	23	21.9		
Field of study					21.76 (3)	<0.001
Physical/biological sciences	18	16.7	27	25.7		
Social sciences	75	69.4	41	39		
Humanities	11	10.2	22	21		
Other	4	3.7	15	14.3		

Socioeconomic status is based upon self-report with 1 = very good and 10 5 = poor.

**Table 2:** Attitudes, support, and religiosity by recruitment site.

Characteristic	OPT		Israel		t (df)	*p* Value
	(*n* = 109)		(*n* = 105)			
	M	SD	M	SD		
ATSPPHS						
Total Score	45.04	8.89	43.7	9.61	1.84 (210)	0.067
Confidence	16.27	4.59	14.36	5.19	2.84 (210)	0.005
Stigma Tolerance	5.38	2.78	5	2.67	1.02 (210)	0.31
Recognition of Need	15.73	4.77	15.27	4.58	0.72 (210)	0.47
Interpersonal Openness	11.91	5.5	13.06	5.52	1.51 (209)	0.13
Perceived Social Support						
Total score	58.78	15.46	64.06	14.53	2.57 (212)	0.011
Family	19.06	6.61	22.77	5.36	4.52 (206.03)	<0.001
Special Friend	21.28	6.43	21.65	6.57	0.42 (212)	0.34
Friends	18.41	6.05	19.58	6.39	1.38 (212)	0.08
Islamic Beliefs	20.94	6.14	19.18	8.71	1.71 (186.29)	0.09
Islamic Belief Practices	25.18	4.32	24.23	5.85	1.35 (190.99)	0.18
Self-rated Health	1.3	0.54	1.3	0.54	0.03 (212)	0.98

For Islamic Beliefs and Islamic Belief Practice, a lower score denotes greater agreement on beliefs and practices (religiosity). Higher scores on the Attitudes toward Seeking Professional Psychological Help Scale and subscales indicate a more positive attitude toward seeking psychological health (with a higher stigma-tolerance score indicating greater tolerance). Higher scores on the Multidimensional Scale of Perceived Social Support total scale and subscales indicate more support.

## Data Availability

Data supporting reported results can be found using the following link: https://drive.google.com/file/d/1JKPcpzEfvi2KWywHJBnz4ed9sDn9eAXy/view?usp=sharing (accessed on 1 July 2022).
